# Analysis of cybersickness in virtual nursing simulation: a German longitudinal study

**DOI:** 10.1186/s12912-024-01833-z

**Published:** 2024-03-20

**Authors:** Maria Biniok, Theresa A. Forbrig, Paul Gellert, Johannes Gräske

**Affiliations:** 1 Department II - Health, Education and Pedagogy, Alice Salomon Hochschule Berlin University of Applied Science, Alice-Salomon-Platz 5, 12627 Berlin, Germany; 2 Institute of Medical Sociology and Rehabilitation Science, Charité– Universitätsmedizin Berlin, Berlin, Germany

**Keywords:** Longitudinal study, Virtual reality, Cybersickness, Nursing education, Skills training, Simulation

## Abstract

**Background:**

Innovative educational approaches such as simulation-based nursing education using virtual reality (VR) technologies provide new opportunities for nursing education. However, there is a lack of information on side effects, especially health-related side effects, of head-mounted displays (HMDs) on the human body when using VR devices for nursing simulation. This study aims to validate the German version of the Virtual Reality Sickness Questionnaire (VRSQ) and to evaluate its associations with sex and age, as reflected in the VRSQ_G_ scores (total score, oculomotor, and disorientation) over time.

**Methods:**

A longitudinal-sectional study was conducted. In addition to the VRSQ_G_ (pre-, post-, and 20 min post-intervention), participants (all nursing students) completed data on personal characteristics. Participants completed a VR simulation of a blood draw. Confirmatory factor analysis (CFA) was used to evaluate whether the measured construct was consistent with the original. In addition to the validity, internal consistency was analyzed and generalized linear models (GLMs) were used for data analysis.

**Results:**

A total of 38 nursing students (mean age 26.8 years; SD = 7.1, 79.0% female) participated. The mean time spent in the VR simulation was 21 min. All participants completed the entire simulation. The CFA indicates (CFI = 0.981, SRMR = 0.040) VRSQ_G_ structure is given. Internal consistency showed low values for the subdomain Oculomotor (Cronbach alpha 0.670). For Disorientation and the Total score values showed a sufficient internal consistency. GLMs showed significant between subject associations with age over time with VRSQ_G_ total score, oculomotor, and disorientation. Older nursing students start with higher VRSQ_G_-Scores. Over time, an approximation occurs, so that all participants reach a similar level by the final measurement point. No associations were found between sex (male/female) and VRSQ_G_ scores.

**Conclusions:**

The VRSQ_G_ is a reliable and valid self-assessment for measuring cybersickness in VR based nursing simulations, with cybersickness symptoms positively associated with age. However, in depth-evaluation regarding age-associations with cybersickness should be done. As well as studies to explore additional associations and emphasizes the importance of establishing cut-off values to assess the clinical relevance of the scores.

**Supplementary Information:**

The online version contains supplementary material available at 10.1186/s12912-024-01833-z.

## Background

Healthcare professionals are facing new challenges, as the complexity of medical and nursing requirements has been increasing for years. This is due to demographic changes, multimorbidity, and new interventions in healthcare. Innovative educational approaches are constantly being developed to address this complexity. One of these approaches is simulation-based nursing education, an established teaching method [1, 2]. Simulation-based nursing is defined as:

*A broad array of structured activities that represent actual or potential situations in education, practice, and research. These activities allow participants to develop or enhance knowledge, skills, and/or attitudes and provide an opportunity to analyze and respond to realistic situations in a simulated environment.* [3]

This method could be applied for undergraduate nursing students as well as experienced nurses [4]. It covers a range of realistic scenarios and spans a continuum from simple, low-fidelity simulations to complex, high-fidelity simulations [5]. High-fidelity simulation is particularly appropriate for teaching because it not only enhances the knowledge and professional skills of nursing students, but also promotes essential clinical competencies such as critical thinking and decision-making, effectively preparing students for practical application in the clinical setting [6]. Learners benefit from a variety of methods, such as role playing and instructor-assigned tasks.

However, simulation with both low-fidelity and high-fidelity manikins has its limitations. The cost of equipment, special requirements, supply planning, the provision of functional care, and patient-related spaces, such as the skills lab simulation room, control room, and debriefing room, are required components [7, 8]. Hybrid forms of augmented reality/virtual reality (AR/VR) platforms and manikin-based simulation models are already known in the literature, and VR simulation technology could offer an alternative approach.

The global trend to integrate VR into education has been growing since the early 1990s [9]. Western countries, including Germany, are now increasingly applying VR trainings in nursing education programs, such as in cross-cultural communication [10] or elder care [11]. Virtual simulations received a boost during the coronavirus pandemic when the International Nursing Association of Clinical Simulation and Learning (INACSL) and the Society for Simulation in Healthcare (SSH) recommended the use of virtual simulation in health professions as an effective teaching method. In addition to clinical hours, virtual simulation aims to enable medical staff to adapt quickly to changing circumstances and continuously improve the quality of patient care [12]. 

VR is a simulated three-dimensional (3D) computer-generated environment crafted to mimic real-world or imaginative scenarios, catering to diverse purposes such as work, education, recreation, and health [13]. This technology utilizes a combination of hardware and software to create an immersive experience for the user, often involving the use of a head-mounted display and other sensory feedback devices. By simulating a realistic environment, VR has the potential to revolutionize various industries. In the field of education, VR can provide interactive and engaging learning experiences, allowing students to explore virtual environments and scenarios that may be impractical or impossible in the real world. They are expected to be more cost effective than traditional skills labs because they require fewer resources [4, 14].

Advanced technologies such as VR are now playing a key role in education. They provide realistic learning environments that can effectively improve the skills and problem-solving abilities of healthcare professionals. They also contribute to minimizing the risk of patient harm and strengthen interprofessional collaboration in the health professions [15, 16]. However, the initial investment is much higher than e.g. live exercises. The substantial initial investment in virtual reality can be distributed to a greater number of trainees over an extended period of time with minimal additional expense, whereas each live drill incurs additional costs that increase in proportion to the number of participants [17]. 

Beside several advantages, in a recently published systematic review, Abbas et al. identified a number of studies reporting motion sickness as an adverse side effect of VR simulations [18]. Motion sickness is a prevalent and intricate syndrome triggered by actual or perceived motion. Traditionally, motion sickness refers to the physical discomfort experienced in real vehicles or during sea voyages, resulting from a mismatch between visual perception and vestibular signals from the inner ear [19, 20]. Its manifestations can vary, encompassing symptoms related to the gastrointestinal system, central nervous system, and autonomic functions [19, 21]. One of the first instruments to measure motion sickness was the Simulator Sickness Questionnaire (SSQ) [22]. The SSQ was originally developed to capture the symptoms of simulator sickness, which is an adaption of motion sickness caused by motions in VR, and it has been used extensively in studies examining the discomfort caused by simulation. As a result, the term *motion sickness* associated with VR has become established in the literature [21, 23, 24]. The evolution of the terms from *simulation sickness* to *motion sickness* and finally to *cybersickness* reflects the technological development and the growing interest in health-related symptoms in virtual environments. This terminological development follows the transition from flight simulator-based applications to more general VR experiences. The key difference between motion sickness and cybersickness is not in the symptoms. While the symptoms of both are similar, the factors triggering them and the environments they appear in are distinct.

In particular, cybersickness is triggered by the discrepancy between perceived movement in virtual environments and the physical stillness of the body in the real world [25–27]. In prior research, approximately 22–80% of participants have reported experiencing cybersickness either during or after using VR applications [28–30]. Symptoms of cybersickness, especially when using screens or other immersive technologies such as HMDs are described as including eye problems such as eyestrain and blurred vision, and more general symptoms such as dizziness, headache, nausea, and general physical discomfort [20, 31, 32]. These symptoms typically occur after 10 min within a simulation [33]. Sensitivity to cybersickness shows considerable individual variability, with some people experiencing minimal provocation while others find it difficult to induce symptoms [34]. Rebenitsch and Owen point out that cybersickness not only affects the user’s well-being, but also poses a safety risk. It can lead to injury or loss of performance [32]. 

As cybersickness can be influenced by a variety of factors, it should be assessed both before and after VR exposure. This approach not only facilitates an understanding of an individual’s baseline symptoms, but also allows for a more accurate determination of the specific effects of VR on cybersickness susceptibility [34, 35]. The SSQ has often been used to measure cybersickness, mainly due to the lack of more specific instruments [36–38]. This realization has led to an increased emphasis on the need to develop and use more specific instruments to measure cybersickness in virtual environments. The Virtual Reality Sickness Questionnaire (VRSQ) is a further development of the SSQ. The items of the VRSQ are therefore based on the SSQ and are used to measure symptoms in the VR environment [39]. The selection of the VRSQ as the measurement instrument in the present study is based not only on its validity and reliability, as highlighted by Sevinc and Berkman, but also on its practicality in application [37]. The VRSQ was chosen because it was specifically designed to capture cybersickness in VR and has been shown to be sensitive to specific movements in these environments. Another key advantage of the VRSQ is its limited number of items. This limitation to a small number of targeted questions makes the VRSQ particularly practical to administer, as it requires less time and is easier for participants to complete. Based on these findings and the specific suitability of the VRSQ for VR, it was selected as the preferred instrument.

Kim et al. use the term motion sickness in their paper based on the SSQ, reflecting the origins and original scope of the questionnaire [39]. In order to provide a more contextually accurate description of the symptoms associated with the use of VR technologies, particularly HMDs, this study adopts the term cybersickness. This shift in terminology not only addresses the specifics of VR environments, but also takes into account the evolving understanding of VR-induced symptoms.

Despite this terminological clarification, there remains a relative paucity of knowledge regarding the prediction of cybersickness in VR use. Recent meta-analyses, including one that found inconsistent results regarding sex and age differences, highlight this gap in understanding [36]. In response, the current study primarily aims to measure cybersickness in the specific context of a nursing-related VR simulation. However, there is no validated German instrument for measuring cybersickness in VR environments. Therefore, the VRSQ firstly was translated into German. Its internal consistency and validity were estimated to ensure that the results are proper. This was followed by an analysis of factors associated with cybersickness.

## Methods

The survey was conducted as part of the Skills.LAB:XR project (4/2020–10/2022). The development and evaluation of VR/XR technologies in the context of simulation-based nursing education was the main objective of the project. In particular, the project focused on the development and evaluation of training scenarios to teach basic skills.

### Sample/participants and settings

The study defined inclusion criteria and required participants to provide voluntary written consent before participating in the research. Participants are nursing students from a University of Applied Sciences and a School for Nursing Professions both in Berlin. All participants had to be at least 18 years old.

Exclusion criteria were applied for the study. Students or trainees who lacked theoretical expertise in the specified research domain were excluded. In addition, individuals with extensive experience in VR within the research field were excluded to maintain a novice level of competence among participants. Other exclusion criteria applied to potential participants with certain pre-existing medical conditions, particularly epilepsy; diagnosed ocular conditions, such as accommodation disorders or spasms; and binocular vision dysfunction.

**Fig. 1:**
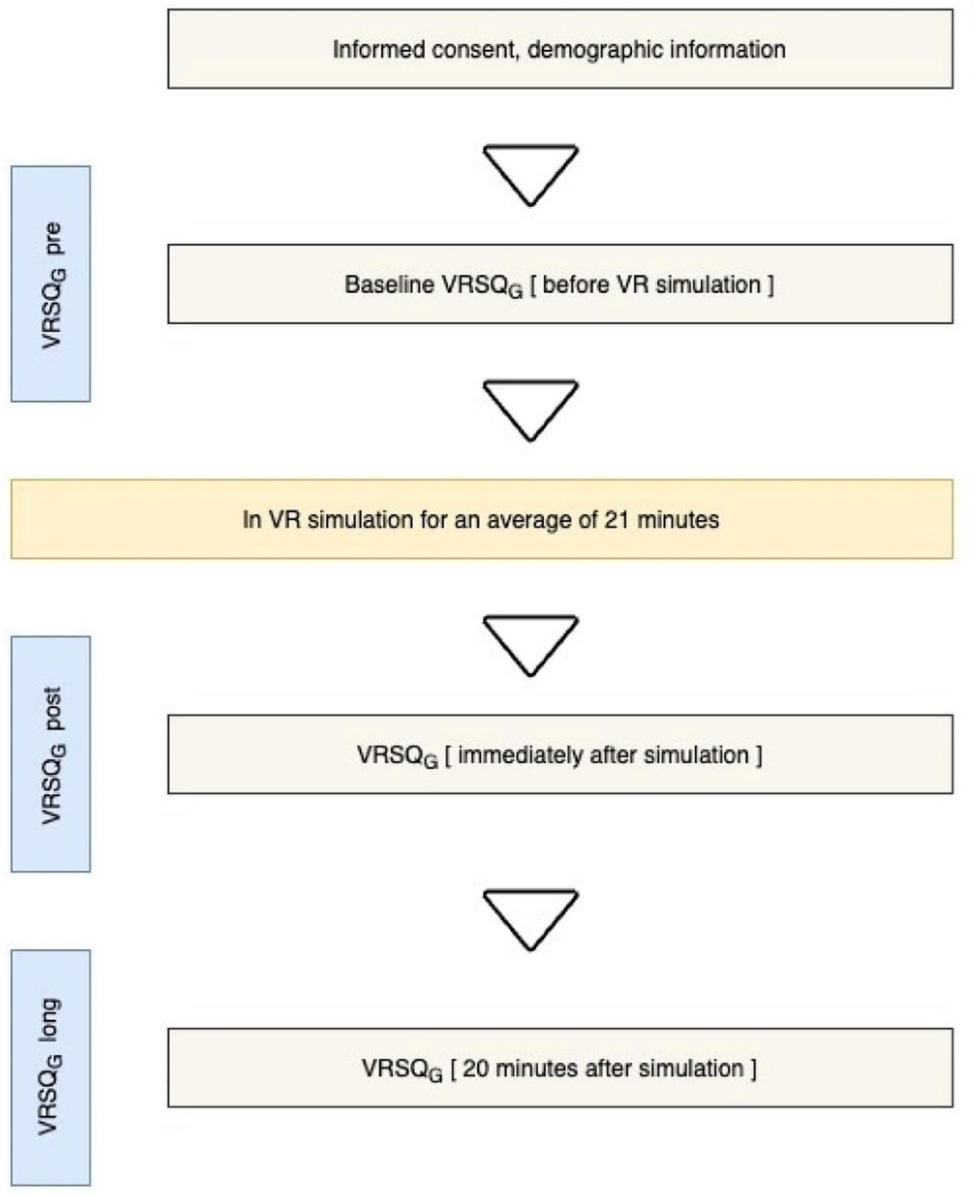
Study design

Four students tested the questionnaire in advance of the data collection. The intervention phase was conducted with a total of 38 students, starting with 22 students in the winter of 2021 and continuing with an additional 16 students from a university of applied sciences in the summer semester of 2022. Prior to beginning the VR simulation, participants received a comprehensive introduction to the research project, including documents such as the project information letter, informed consent form, and data protection sheet (see Fig. 1). This was followed by the first administration of the VRSQ_G_ using the QUAMP software (v 4.5.8), an online survey tool. To determine individual predispositions to cybersickness prior to entering the VR environment, pre-trial data were collected focusing on three main categories: demographic information, health and fitness status, and known cybersickness triggers [40]. In the next step, participants were instructed on how to use the HMD (HTC Vive Pro Eye) and the Manus-Prime data gloves (MANUS Prime X Haptic VR).

### Intervention

Only one participant was in the VR environment at any given time, and the intervention occurred in a typical classroom setting where the VR simulation was displayed. After putting on the HMD and gloves and being onboarded, the participants had the opportunity to explore the virtual skills lab and take their first steps. Participants collected information from the virtual patient record and the physician’s procedure instructions and performed an initial anamnesis with the virtual patient avatar (MetaHuman). In the virtual environment, patient avatars were created with different sex and physical appearances, complete with facial animations to convey their unique characters through expressive gestures and facial expressions. The facial animations were integrated into the game engine, allowing each avatar to have up to 10 different expressions, such as calm, curious, anxious, and nervous, which could be activated externally at the touch of a button. The behavior of the avatars was further enhanced with different sitting and lying positions. Steps such as disinfection, material preparation, patient data verification, and blood collection are practiced in the virtual simulation. For this purpose, 3D objects required for venous blood collection are used. These specific objects, including packaging, glove boxes, adapters, and butterfly needles, are designed to be operated interactively with the gloves. This integration of detailed, realistic elements aims to replicate the tactile and procedural aspects of nursing tasks, enriching the immersive learning experience. The training scenario, including all its nursing-specific details, was developed using Unreal Engine 4.26 within the framework of the research project.

A research assistant took on the role of the patient’s voice and switched the avatar into a “speaking mode” in which spoken words (via microphone input) were synchronized with the avatar’s mouth movements, audible through the VR headset’s headphones. The participants assembled the materials needed for the venous blood collection and performed the procedure, partly under the verbal guidance of the researchers. Participants received guidance and supervision from two experienced nurses and research project staff, as well as a technical expert in game design.

The support for the action steps was based on the participants’ previously collected theoretical and practical experience. The learning status was recorded by self-assessment (e.g., Do you have prior knowledge of blood collection? (1) From theoretical classes; (2) From practical lessons; (3) From the practice). The aim was to keep the duration of the simulation in VR to at least 20 min, as symptoms of cybersickness can increase after this time [41]. On average, participants spent approximately 21 min in the virtual simulation (see Fig. 1). All participants completed the entire simulation, so there were no dropouts.

### Instrument

#### Translation and cultural adaptation

The process of translating and culturally adapting the VRSQ to German required approval from principal author from Incheon National University (Rebublic of Korea). The translation process followed the guidelines for translating a questionnaire into another language by Tsang et al. [42]. The translation and cultural adaptation process consisted of five distinct phases. In phase 1, translation, the material underwent two separate forward translations into German. In phase 2, synthesis, a committee of experts discussed any discrepancies until consensus was reached. Phase 3, back translation, involved a reverse translation without reference to the original text, which served as an essential proof of concept. In phase 4, original author approval, the back-translated version was reviewed and approved by the author of the original material. Finally, phase 5, pretest, was conducted: The German version of the VRSQ was pre-tested by four students in order to identify any problems with understanding or answering the questionnaire items.

At the beginning of the questionnaire, various personal characteristics (e.g., age, sex) were recorded. Cybersickness was evaluated using the VRSQ, which has already been published by Kim et al. [39]. This instrument comprises two subdomains (oculomotor, disorientation), with a total number of nine items (see Table 1).

**Table 1:** Items of the VRSQ

VRSQ symptom	Oculomotor	Disorientation
1. General discomfort	X	
2. Fatigue	X	
3. Eyestrain	X	
4. Difficulty focusing	X	
5. Headache		X
6. Fullness of head		X
7. Blurred vision		X
8. Dizzy (eyes closed)		X
9. Vertigo		X
Total	Sum [1]	Sum [2]

Each item is rated on a Likert scale of 0 (not at all), 1 (slightly), 2 (moderately), 3 (very strong). Calculations of the domain and total scores are displayed in Table 2.

**Table 2:** Scoring

Components	Computation
Oculomotor	([1]/12)*100
Disorientation	([2]/15)*100
Total	(Oculomotor score + Disorientation score) / 2

### Data collection

All participants completed the questionnaire at three fixed times before the simulation, immediately after the VR simulation, and 20 min after completion of the VR simulation using QUAMP software. Participants completed the online questionnaire in a separate room to avoid interaction with other participants.

### Data analysis

In a first step, data cleaning regarding outlier and illogical data was performed. To describe the data, typical parameters such as mean and standard deviation (SD) were used. To analyze association with pre-scores of VRSQ_G_ Oculomotor, VRSQ_G_ Disorientation, and VRSQ_G_ Total, ANCOVA models were analyzed. Influencing factors were sex (male/female) and covariate age in years. To analyze the development over time, generalized linear models (GLMs) were used. Dependent variables were the pre-, post-, and long scores of VRSQ_G_ Oculomotor, VRSQ_G_ Disorientation, and VRSQ_G_ Total. Influencing factors were again sex (male/female) and covariate age in years. After data cleaning, data description was conducted using typical parameters, such as mean, SD, and absolute and relative numbers.

### Internal consistency

Internal consistency was estimated using Cronbach`s alpha. This measure indicates how well items of the same latent variable measure this variable. A good sufficient internal consistency is assumed with values above 0.7 [43]. 

### Validity

To estimate the structure of the VRQS_G_, a confirmatory factor analysis (CFA) was performed, using R 4.2.2 (including lavaan package). Hu and Bentler recommend a two-index representation of results of CFA. For small samples (< *n* = 250), the comparative fit index (CFI) should be above 0.95 and the standardized root mean square (SRMR) less than 0.06 [44]. To minimize the quantity of manifest parameters and a sample item, parceling was used to reduce the quantity of manifest parameters and to increase stability parameter estimates. Each parcel represents the mean value of at least two items. This estimate is the new indicator of a latent construct. Each item was allocated to a parcel regarding its content. The allocation was conducted with respect to incorporate a heterogeneity in each parcel. Data description and analysis were conducted with SPSS^®^ 28. The CFA was conducted using R 4.2.2 (including lavaan package). All tests were interpreted regarding a significance level of 0.05.

## Results

### Translation and cultural adaptation

**Fig. 2:**
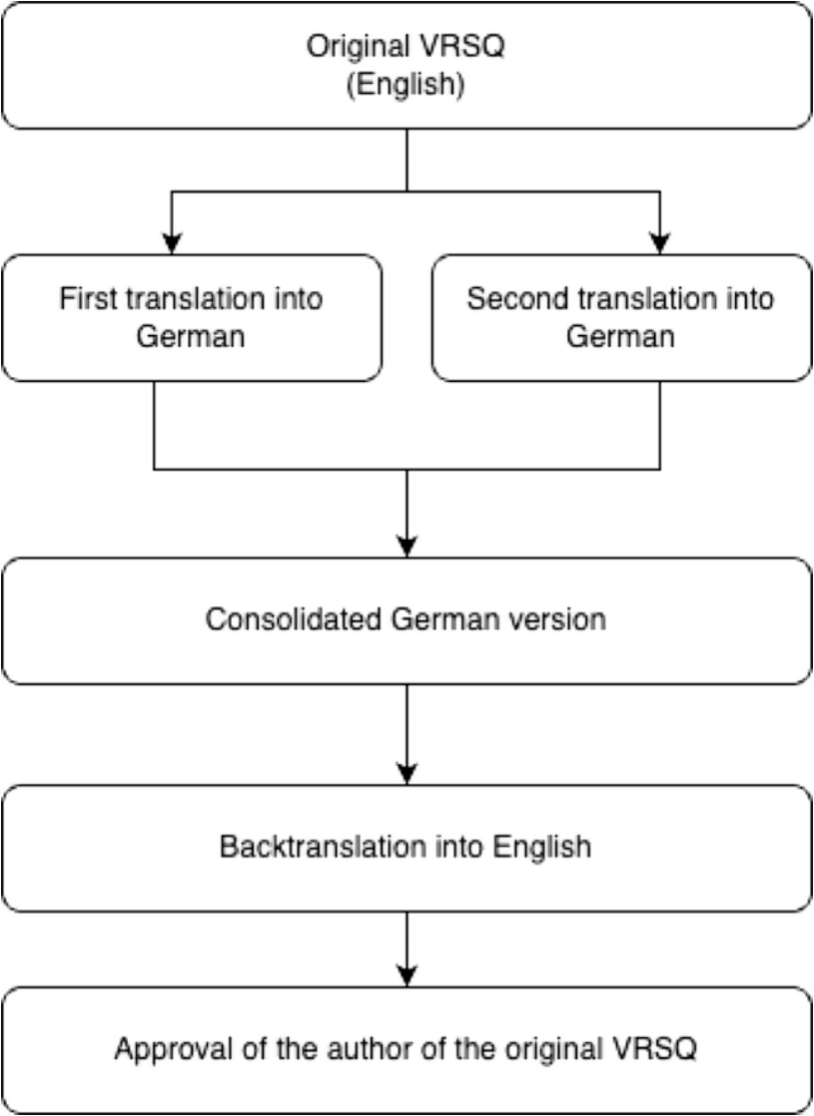
Translation process

The translation and cultural adaptation process is described in detail in the Methods section. This five-phase process resulted in a consistent German version of the VRSQ (see Fig. 2).

#### Phases 1 and 2: translation and synthesis

The translations from English to German were largely consistent. Differences were found in the items *fatigue* and *exhaustion*, in the item *fullness of the head*, and in the equivalent content of the items *dizzy (eyes closed) / dizziness (with eyes closed)* and *vertigo*. The differences were discussed within the research team, and a linguistic choice was made. This resulted in a consolidated German version of the VRSQ (VRSQ_G_).

#### Phase 3: back translation

The consolidated German version was back translated into English by a native English translator. The professional translator was not familiar with the original version. The back translation was a necessary step to check the accuracy of the translation. It involved translating the material from the target language (German) back to the source language (English).This process helps to uncover any misunderstandings in the initial translations [42]. 

#### Phase 4: expert committee review

The current authors reviewed the back-translated version in detail and found no discrepancies between it and the original version. This back-translated document was then submitted to the developer of the original version for detailed review, as shown in Fig. 2. Approval was then obtained from the principal author of the original instrument.

#### Phase 5: pre-testing

Before starting the validation process, a pre-test phase was conducted in which four students participated. This pre-test phase was used to identify potential problems with the items. During this phase, the students interacted with the items, and their responses and feedback were carefully observed and recorded. In addition, all four students gave their impressions of the items in a personal interview. Unclear items were discussed, and suggestions for improvement were evaluated. The results of this preliminary evaluation showed that the students had almost no difficulties in understanding or answering the items. This result shows that the questionnaire is appropriately designed for the target group. A total of 38 participants with an average age of 27 years participated in the project, 78.95% of whom identified as female (see Table 3).

**Table 3:** Sample characteristics (*n* = 38)

	Total (*n* = 38)
**Age** in years, mean (SD)	26.87 (6.99)
**Sex**, % (n)	
Female	79.0 (30)
Male	21.1 (8)

### Reliability

The estimates of Cronbach’s alpha indicate a sufficient internal consistency for the subdomain disorientation and the total score (see Table 4). However, the oculomotor values are a bit lower than expected.

**Table 4:** Reliability of the VRSQ_G_

Domain	Cronbach’s alpha
VRSQ_G_ - Oculomotor	0.670
VRSQ_G_ - Disorientation	0.759
VRSQ_G_ - Total	0.822

### Validity

The CFA of the VRSQ_G_ (see Table 5) indicates sufficient to good model fits. CFI = 0.981 and SRMR = 0.040 resulted in values in the preferable range.

### Associations with the VRSQ_G_

**Table 5:** Association with cybersickness

	VRSQ_G_ - Oculomotor			VRSQ_G_ - Disorientation			VRSQ_G_ - Total		
	b	p-value	ηp2	b	p-value	ηp2	b	p-value	ηp2
**Pre**									
Corr. model		0.041	0.167		0.039	0.169		0.042	0.165
Constant term	6.615	0.518	0.012	-2.154	0.826	0.001	2.230	0.805	0.002
Male^*^	3.298	0.599	0.008	-10.566	0.085	0.082	-3.634	0.513	0.012
Age^#^	**0.936**	**0.014**	**0.160**	**0.711**	**0.048**	**0.107**	**0.824**	**0.015**	**0.158**
**Post**									
Corr. model		0.845	0.010		0.191	0.090		0.540	0.035
Constant term	20.696	0.085	0.083	**20.646**	**0.004**	**0.111**	20.671	0.053	0.103
Male^*^	-3,203	0.658	0.006	-10.988	0.079	0.085	-7.095	0.270	0.035
Age^#^	0.161	0.703	0.004	− 0.140	0.695	0.004	0.010	0.978	0.000
**Long**									
Corr. model		0.738	0.017		0.292	0.068		0.628	0.026
Constant term	**28.240**	**0.024**	**0.137**	11.998	0.112	0.071	**20.119**	**0.033**	**0.124**
Male^*^	-2.143	0.772	0.002	-7.226	0.119	0.068	-4.685	0.406	0.020
Age^#^	− 0.308	0.477	0.015	− 0.008	0.975	0.000	− 0.159	0.648	0.006

Pre: directly before the simulation starts; post: directly after the simulation ended; long: 20 min after the simulation ended

Bold values are significant to α = 0.05

*Reference category: female

^#^Age in years: co-variable

The analysis of associations between sex and age with VRSQ_G_ Oculomotor, VRSQ_G_ Disorientation, and VRSQ_G_ Total Score of all three time points does not show a significant association for sex at any time point (all *p* >.05). However, age in years is positively associated with all pre-simulation VRSQ_G_ scores (*p* <.05, b > 0.711).

**Table 6:** Associations with cybersickness over time

	Within-subject effects			Between-subject effects		
		p-value	ηp2	(co-)variable	p	ηp2
VRSQ_G_– Oculomotor	Time	0.282	0.036	Constant term	0.056	0.100
	Time*sex	0.623	0.013	Sex	0.903	0.000
	Time*age	**0.014**	**0.115**	Age	0.424	0.018
VRSQ_G_– Disorientation*	**Time**	**0.023**	**0.110**	Constant term	0.507	0.013
	Time*sex	0.654	0.011	Sex	0.055	0.101
	Time*age	**0.010**	**0.133**	Age	0.498	0.013
VRSQ_G_– Total*	Time	0.096	0.068	Constant term	0.160	0.056
	Time*sex	0.753	0.006	Sex	0.307	0.030
	Time*age	**0.008**	**0.142**	Age	0.435	0.017

*Greenhous–Geisser

Bold values are significant to α = 0.05

Looking at the longitudinal associations for all three VRSQ_G_ scores (see Table 6), an effect for time in combination with age was found (all *p* <.05). Additionally, an effect for time was found for VRSQ_G_ Disorientation (*p* =.023).

**Fig. 3:**
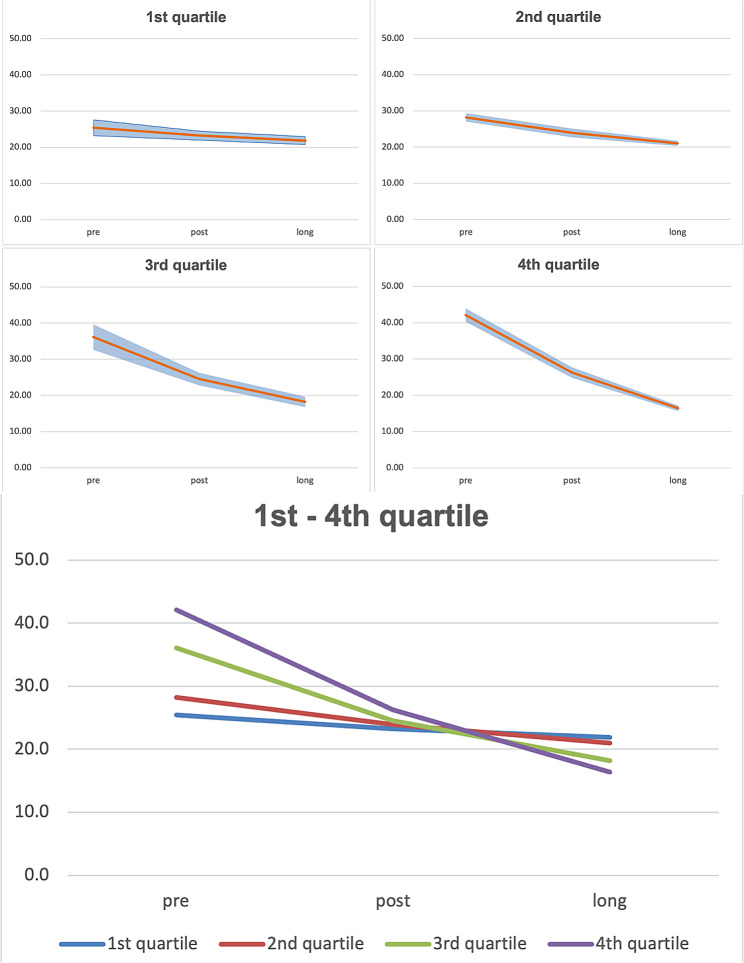
Predicted VRSQ_G_– Oculomotor for the each age related quartile

Figure 3 shows, that VRSQ_G_-values remain quite stable over time for the 1st and 2nd age quartile. While the 3rd and 4th age quartile start with quite high scores and decreasing values over time.

**Fig. 4:**
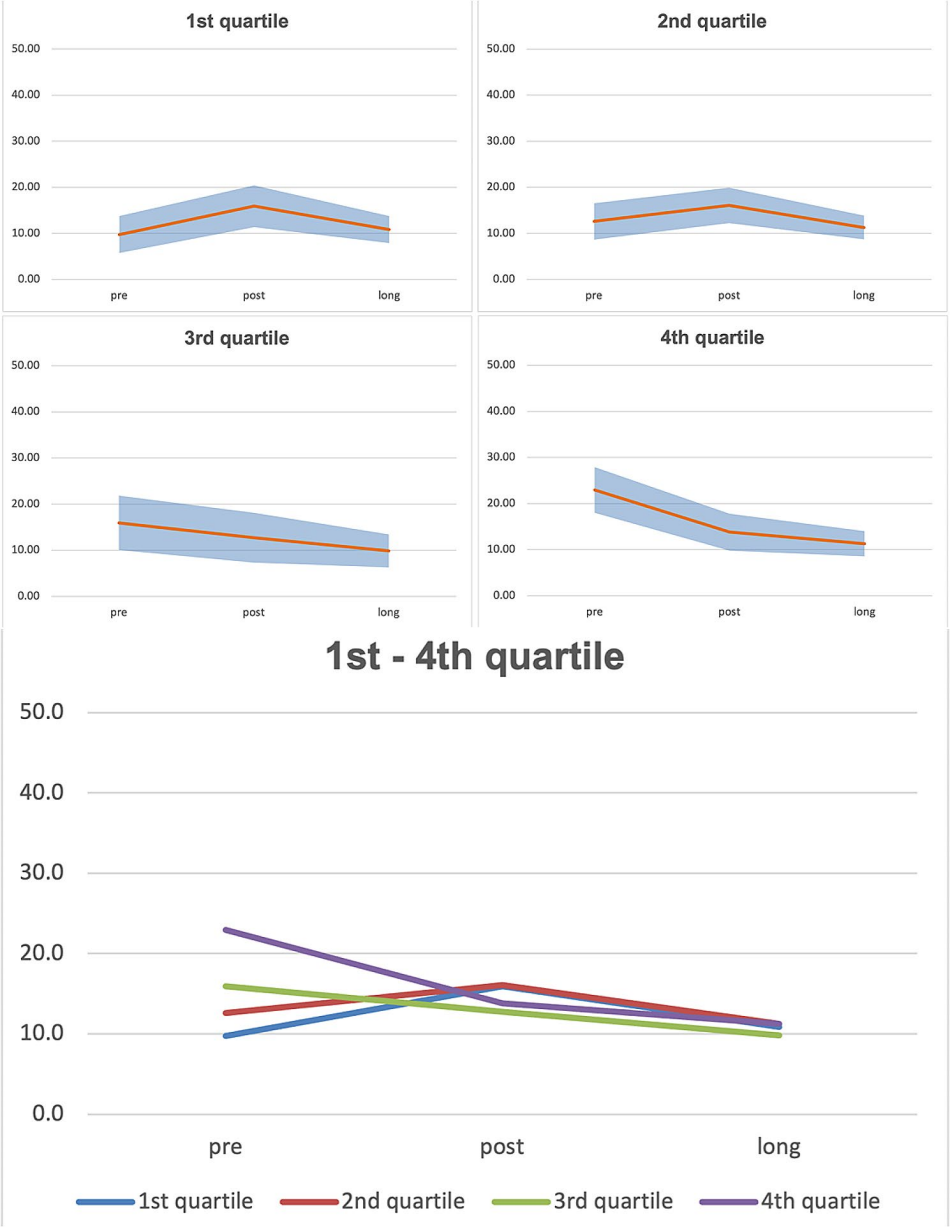
Predicted VRSQ_G_– Disorientation for the each age related quartile

In Fig. 4 values of VRSQ_G_ Disorientation are shown. The 1st and 2nd age quartiles show increased values for the post time point and quite similar values for the pre and long time points. However, for the 3rd and 4th age quartiles, decreasing values over time are predicted.

**Fig. 5:**
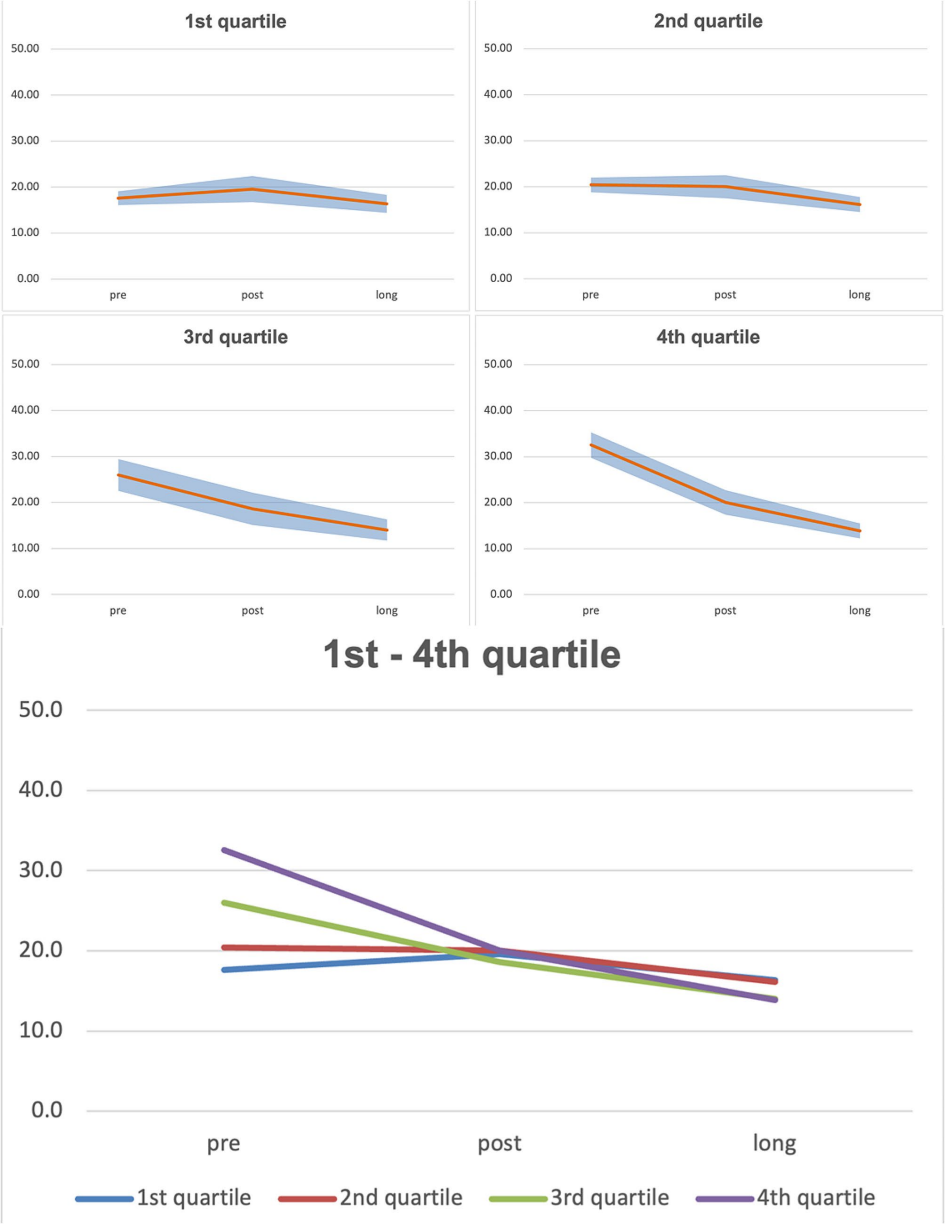
Predicted VRSQ_G_– Total for the each age related quartile

Figure 5 displays values of VRSQ_G_ Total while values for the 1st and 2nd age quartiles slightly decrease over time, for the 3rd and 4th age quartiles a greater decrease of predicted values are shown.

**Fig. 6:**
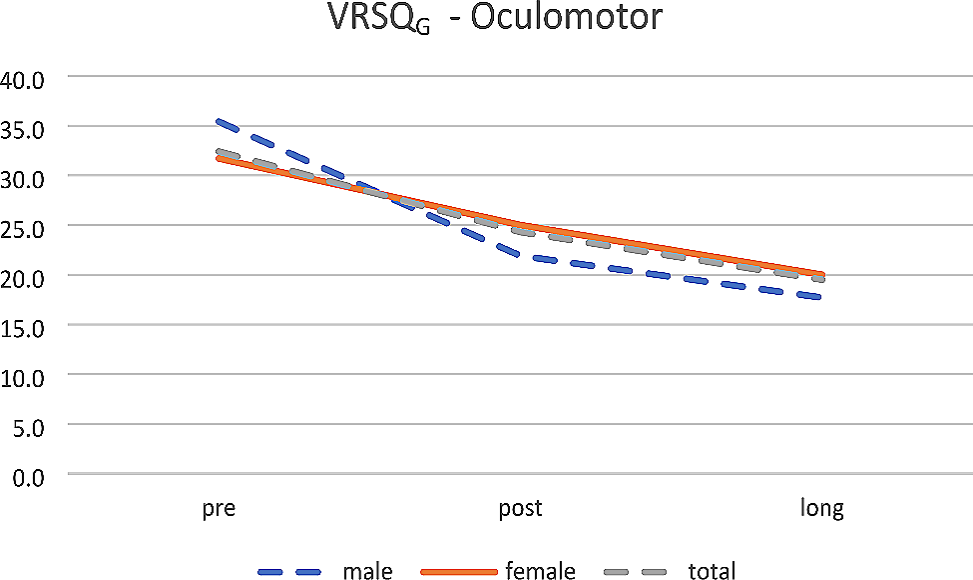
VRSQ_G_– Oculomotor (mean age 26.8 years)

**Fig. 7:**
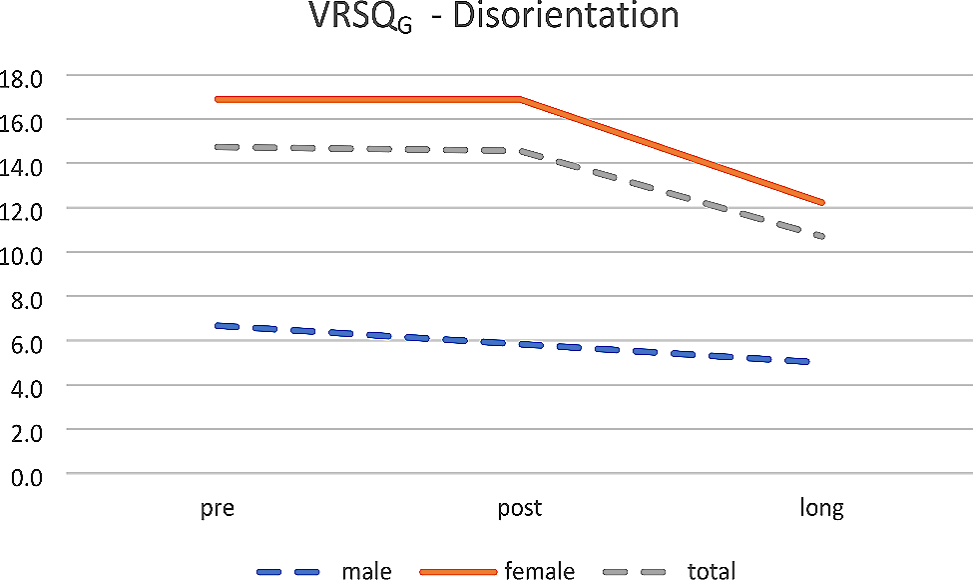
VRSQ_G_– Disorientation (mean age 26.8 years)

**Fig. 8:**
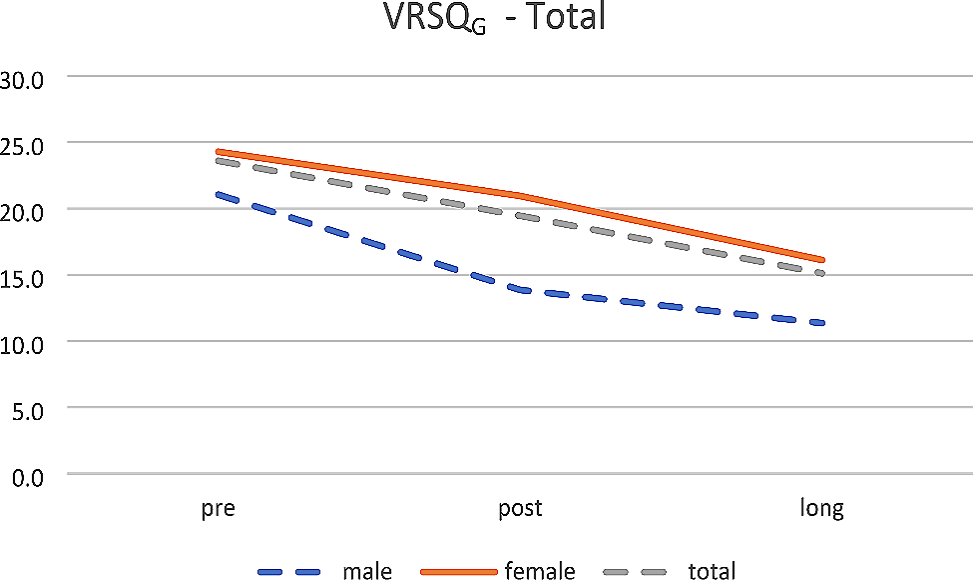
VRSQ_G_– Total (mean age 26.8 years)

Not any effect was found for sex. Anyway, the scores decrease over time for oculomotor (see Fig. 6), disorientation (see Fig. 7) and total (see Fig. 8).

## Discussion

With a particular focus on the role of VR in nursing education and the challenges associated with it, such as cybersickness, this section highlights how the study findings extend current knowledge and what implications they have for the future application of VR in educational settings. Age and sex distribution of the present study participants differ from those in the other studies. Participants are about three years older than those in the study of Kim et al. [39]. And the proportion of female participants is higher (79.0% vs. 50.0%).

Kim et al., who developed the VRSQ, had very high internal consistency scores, indicating excellent item consistency [39]. The VRSQ_G_ tested in this study had sufficient Cronbach’s alpha values, referring to a good internal consistency for the domains of disorientation and total score (all > 0.7). The oculomotor subdomain has slightly lower Cronbach’s alpha values (0.670) than expected, so the items in this specific score of the questionnaire are not as consistent as preferred. The study by Sevinc and Berkman shows overall high internal consistencies in all domains of the VRSQ [37]. Kourtesis et al. show similar results of the original version compared to the current results of the VRSQ_G_, with acceptable to good internal consistency, but slightly lower scores on the oculomotor subscale [38]. The testing of the validity showed preferable results, so it can be concluded that the VRSQ_G_ can be considered as a valid instrument.

Differences in the results of psychometric testing of the VRSQ may be due to several factors. Even though the VRSQ_G_ was approved by the author of the original instrument, translation and cultural differences may have changed the interpretation of the items. This may also occur when the instrument is used and validated in other languages and cultures, such as Turkish. Similarly, demographic differences within the sample (age, education level, previous experience with VR) or the area of application (clinical environment, educational institution, leisure use) as well as the technical equipment (HMDs, smaller screens) within the studies may influence the results. It is recommended that the latest VR software and external hardware be used, as this will reduce health and safety risks to participants and may also increase the reliability of results, for example, by reducing dropout rates [36, 45]. Considering that demographic differences, application area, and technical equipment may influence the results, it is recommended that the questionnaire has sufficient internal consistency overall. The lower scores in the oculomotor domain indicate the need for further research in this subdomain to improve the reliability of the questionnaire.

Sanchez et al. also see major challenges related to VR and research in the reliability and validity of assessments. They cite the dynamic characteristics and multiple interactive functions within a VR simulation. It can be assumed that participants will repeatedly report differences in their perceptions, experiences, and thus results over time. This can lead to measurement error compared to traditional assessments and questionnaires [46]. Based on the fact that even with improvements in VR technology [45], some participants still suffer from cybersickness [32], Sanchez et al. recommend that future research use scales such as the VRSQ to measure cybersickness and incorporate the results into their evaluations and, for example, further developments of VR simulations.

The focus is on the analysis of the relationship between sex, age, and VRSQ_G_ scores. The analysis shows that there is no significant correlation between participants’ sex and VRSQ_G_ scores in the oculomotor, disorientation, and total categories at any of the three measurement points (all *p* >.05). However, it is striking that age in years is positively associated with all pre-simulation VRSQ_G_ scores (*p* <.05, b > 0.711). This reveals in a stronger decreasing value for higher age groups than for younger nursing students.

Regarding the different results of the meta-analysis by Saredakis et al. and Howard and Van Zandt, as well as the study by Garrido et al., the present results suggest that sex is not while age is positively associated with cybersickness [34, 36, 47].

The observed positive association between participants’ age and pre-simulation VRSQ_G_ scores suggests that older individuals may be more susceptible to cybersickness, indicating the instrument’s effectiveness in capturing age-related perceptual differences. It also underscores the importance of considering age in the design of VR content, particularly for educational purposes, and highlights the need for research focused on age-related changes in perception and balance. Furthermore, these findings emphasize the need to develop tailored strategies for adapting VR learning to mitigate cybersickness in older users, and they invite further research on VR habituation effects. Such research could improve the overall effectiveness and acceptance of VR applications among different age groups. In addition to further research on adapted strategies, a broader look at a more differentiated categorization of age is needed. Based on the available data, Garrido et al. and Howard and Van Zandt conclude the following: it is possible that the relationship between these variables is non-linear throughout life, suggesting that children and older adults may experience more cybersickness than younger and middle-aged adults [34, 47]. 

Looking at the longitudinal associations across all three VRSQ_G_ scores (see Table 6) reveals a significant influence of time and age on cybersickness experiences in VR (all *p* <.05). The decreasing trend in VRSQ_G_ scores, especially for older nursing students, over time suggests that individuals may adapt to the VR environment, underscoring the ability of the VRSQ_G_ to capture changes in the experience of cybersickness over time. It can also be assumed, that younger nursing students may have had more contact with VR simulations before. They might be more accustomed to the VR technology than older nursing students. A meta-analysis of factors associated with cybersickness found a correlation between exposure time spent in VR and scores on the nausea and disorientation subscales of the original SSQ, the predecessor to the VRSQ. Scores varied depending on whether participants spent more or less than 10 min in the VR environment. A meta-analysis [36] of factors associated with cybersickness found an association between time spent in VR and symptoms of cybersickness. The scores varied nonlinearly over time.

While an initial peak in symptoms is observed, longer durations (over 20 min) indicate adaptation to content and/or image sequences and may reduce symptoms [36]. The ability to capture such changes may prove useful in understanding how individuals adapt to VR over time. The specific time effect on VRSQ_G_ disorientation further suggests that certain aspects of cybersickness, such as disorientation, are more susceptible to change over time. Taken together, these findings suggest that the experience of cybersickness in VR is dynamic, influenced by both individual factors (e.g., age) and the duration of exposure. However, the understanding of side effects, like cybersickness, of VR simulations in nursing education, contributes to a broader application.

### Limitations

A pragmatic sampling approach was used in this study. Due to the nature of participant recruitment, which was based on voluntary attendance during the courses, it was not possible to determine a response rate. Despite a comprehensive recruitment strategy, sampling bias cannot be completely ruled out. The small number of participants means that the results should be generalized with caution, especially as they do not reflect the participants in the original study in terms of age and sex distribution. The scores on the VRSQ_G_ may have been affected by the small size of the participant group. In addition, the sample consisted of young adults, so no conclusions can be drawn about a broader age range of participants. Future studies should evaluate a sample with a broader age range. It is possible that participants rated their symptoms based on previous experience. These issues must be considered when interpreting the results, particularly with regard to the use and effectiveness of the VRSQ_G_.

## Conclusions

The results of the present study confirm that the VRSQ_G_ is a reliable and valid self-assessment to measure cybersickness in VR environments, with a positive association between cybersickness symptoms and age. Further research is needed to examine additional variables (e.g., demographic factors and individual differences) that may influence cybersickness. Due to the dynamic field of VR learning, in-depth studies need to be conducted to determine further associations so that VR experiences can be tailored to individual needs. In addition, it is important to evaluate the long-term effects of VR exposure to understand whether users may develop habituation or sensitization to cybersickness over time. However, it remains unclear whether the scores have clinical relevance. Cut-off values should be established.

### Electronic supplementary material

Below is the link to the electronic supplementary material.

VRSQ - Original and German Version

## Data Availability

The datasets analyzed during the current study are available from the corresponding author on reasonable request.
