# Correction: Analysis of cybersickness in virtual nursing simulation: a German longitudinal study

**DOI:** 10.1186/s12912-024-01971-4

**Published:** 2024-05-28

**Authors:** Maria Biniok, Theresa A. Forbrig, Paul Gellert, Johannes Gräske

**Affiliations:** 1 Department II - Health, Education and Pedagogy, Alice Salomon Hochschule Berlin University of Applied Science, Alice-Salomon-Platz 5, 12627 Berlin, Germany; 2 Institute of Medical Sociology and Rehabilitation Science, Charité– Universitätsmedizin Berlin, Berlin, Germany

**Correction to: BMC Nursing (2024) 23:187**

10.1186/s12912-024-01833-z

Following publication of the original article [1], the authors reported one typing error in Table 2, line Disorientation is written: ([2]/12)*100 but it should be: ([2]/15)*100.

**Table 2:** Scoring

Components	Computation
Oculomotor	( [1]/12)*100
Disorientation	([2]/15)*100
Total	(Oculomotor score + Disorientation score) / 2

The correct Table 2 has been provided in this Correction.

Furthermore, the authors were informed by Dr. Berkmann although their study (Sevinc V, Berkman MI. Psychometric evaluation of Simulator Sickness Questionnaire and its variants as a measure of cybersickness in consumer virtual environments. Appl Ergon. 2020;82:102958) was conducted in Turkey, they applied the English version of the VRSQ. Therefore the following sentence in the discussion section has to be corrected as well: The study by Sevinc and Berkman shows overall high internal consistencies in all domains of the VRSQ [37].

The original article [1] has been corrected.
