# Supplementary material for: Analysis of cybersickness in virtual nursing simulation: a German longitudinal study
Source: BMC Nurs. 2024 Mar 20;23:187. doi: 10.1186/s12912-024-01833-z (PMC10953248; doi:10.1186/s12912-024-01833-z)
Supplement: Supplementary file 1 — VRSQ - Original and German Version [file 12912_2024_1833_MOESM1_ESM.pdf]

Virtual Reality Sickness Questionnaire (VRSQ) by Kim et al.

	0 (= not at all)	1 (= slightly)	2 (= moderately)	3 (= very)
1. General discomfort				
2. Fatigue				
3. Eyestrain				
4. Difficulty focusing				
5. Headache				
6. Fullness of head				
7. Blurred vision				
8. Dizzy (eyes closed)				
9. Vertigo				

Kim HK, Park J, Choi Y, Choe M (2018) Virtual reality sickness questionnaire (VRSQ): Motion sickness measurement index in a virtual reality environment. Applied Ergonomics 69, 66–73

German version of Virtual Reality Questionnaire ($VRSQ_G$)

	0 (= gar nicht)	1 (=leicht)	2 (=mäßig)	3 (=sehr)
1. Allgemeine Beschwerden				
2. Müdigkeit				
3. Überanstrengung der Augen				
4. Schwierigkeit beim Fokussieren				
5. Kopfschmerzen				
6. Druckgefühl im Kopf				
7. Verschwommenes Sehen				
8. Schwindel (Augen geschlossen)				
9. Schwindel				

German version with the kind permission of Dr. Jaehyun Park: © 2024 $VRSQ_G$.
Maria Biniok, RN, MSc, Paul Gellert, PhD, Theresa A. Forbrig, RN, MSc, Johannes Gräske, RN, PhD, Alice Salomon Hochschule Berlin, Alice-Salomon Platz 5, 12627 Berlin, biniok.graeske@gmail.com.
Non-commercial use permitted. Any form of commercial use, such as by reprint, sale, or electronic publication, requires prior written permission, as does distribution through electronic media.
